# Revolutionizing Tissue Clearing and 3-Dimensional Imaging: Transparent Embedding Solvent System for Uniform High-Resolution Imaging

**DOI:** 10.34133/bmef.0095

**Published:** 2025-01-23

**Authors:** Yating Yi, Hu Zhao

**Affiliations:** ^1^ State Key Laboratory of Oral Diseases, National Center for Stomatology, National Clinical Research Center for Oral Diseases, West China Hospital of Stomatology, Sichuan University, Chengdu, Sichuan, China.; ^2^ Chinese Institute for Brain Research, Beijing, China.

## Abstract

Combining transparent embedding with sectioning is likely to be the future direction for tissue clearing and 3-dimensional (3D) imaging. A newly published transparent embedding system, TESOS (Transparent Embedding Solvent System), ensures consistent submicron resolution imaging throughout the entire sample, and can be compatible with different microscopy systems. This method shows great potential in connectome mapping, and might be an optimal option for future 3D multiplex immunofluorescence and RNA in situ hybridization imaging. Additional efforts would be needed to innovate labeling, imaging, and data processing strategies to fully utilize the potential of transparent embedding systems in high-resolution imaging of large-scale samples.

As the demand for studying complex biological structures grows, 3-dimensional (3D) optical imaging has gained great attention from researchers. This technique offers a clear view of the inner workings of tissues and organs, helping us better understand anatomical complexities, particularly in the nervous system. 3D imaging is crucial for unbiased exploration of entire organisms and neural networks in mammals, making it a key objective in biological research.

One of the challenges in large-volume 3D imaging is light scattering in the deeper layers of tissues due to their opacity. The development of tissue clearing techniques has been a breakthrough in overcoming this obstacle and enabling effective 3D optical imaging. Currently, there are 3 main types of tissue-clearing methods: hydrophobic, hydrophilic, and hydrogel-based tissue clearing method [1–6]. Although these methods use different chemicals and strategies, they generally operate on similar chemical principles [7,8]. By removing components like calcium phosphate, pigments, lipids, and water (in the case of hydrophobic methods) and matching the refractive indices of the sample and imaging media, tissues can become transparent and suitable for imaging with light sheet or confocal microscopy. This allows for the imaging of whole organs, including brains from rodents and nonhuman primates, as well as entire rodents after tissue clearing.

However, achieving consistently high resolution throughout the entire sample, especially for large samples like the whole body of an adult mouse, remains a challenge for all tissue clearing methods. Signal intensity and resolution can drop substantially in the deeper regions [9]. This is due to inadequate tissue transparency, imperfect matching of refractive indices [10], and, more importantly, the limitations of existing imaging technologies. As a result, none of the previous methods have been able to fully map the projection of individual neurons from the peripheral nervous system (PNS) to the central nervous system (CNS) in a rodent model.

Recently, Yi et al. [11] introduced a new method called the Transparent Embedding Solvent System (TESOS) in *Cell Research*. This method combines tissue clearing, transparent embedding, sectioning, and block-face imaging to address the challenges mentioned earlier and allows for imaging large samples with consistent high resolution.

After a pre-treatment, samples are soaked in a clearing solution that contains benzyl benzoate and bisphenol-A ethoxylate diacrylate Mn 468, both of which have a high refractive index, along with an ultraviolet (UV) crosslinking initiator. The cleared samples and the clearing solution are then exposed to UV light for several minutes, which helps form an organogel that has good mechanical strength. The curing setup and time need to be optimized according to the type and size of samples. For a mouse brain sample, curing duration is about 5 min; for large samples including a mouse pup or adult mouse body trunks, customized glass chambers are used to allow UV light coming from all angles. This process could accommodate samples as large as an adult mouse whole body, which can be polymerized within 30 min. The samples within the organogel stay transparent, and their endogenous fluorescence is preserved after treatment.

Next, the samples are attached to the upper part of a MagMount device. The device’s bottom plate is placed on the sample movement stage beneath the microscope, while another bottom plate is set up on a rotary microtome. The samples are imaged using a confocal microscope, with the imaging thickness aligned to the objective’s working distance. After this, the samples are moved to the microtome to remove the top 90% of the section that was imaged. All the acquired image stacks are then stitched together for further 3D data analysis. For whole-body imaging of an adult mouse, ~70 terabytes of data for 2 channels were generated. A customized software pipeline was developed to implement automated volume stitching, which took about 1 week to complete accurate stitching using the high-performance computing (HPC) platform mentioned in the article.

The TESOS method can be used with different microscopy systems. The team successfully imaged the whole body of an adult mouse at a uniform micrometer voxel resolution using TESOS combined with light sheet microscopy. They achieved submicron resolution imaging for large tissue samples with confocal microscopy and presented the first complete mesoscale connectome mapping of individual sensory nerve axons from the PNS to the CNS.

3D imaging at microscale resolution is crucial for mapping neuron connectivity and understanding the molecular features of cells [12]. In the current realm of optical microscopy, there is a fundamental trade-off among imaging resolution, sample size, and the cost of imaging [13]. Objectives with high numerical aperture (NA), which provide high resolution, typically have a short working distance, limiting how deep they can image [14]. Some studies have reported customized objectives that combine high NA with long working distance for imaging cleared samples, but these are expensive and require specific imaging systems [13,15–17]. Additionally, due to the insufficient transparency of tissue samples, minor optical aberrations can always occur and accumulate along the optical path, leading to inevitable resolution loss in deeper regions. Transparent embedding overcame the above challenges and achieved uniformly high-resolution 3D imaging in large samples. By integrating sectioning into the tissue clearing and 3D imaging processes, researchers can easily address the limitations of working distance associated with optical objectives. This allows for the selection of commercial objectives to obtain high-quality images, regardless of working distance and sample thickness.

Both the TESOS method and section-reconstruction imaging techniques like fluorescence micro-optical sectioning tomography (fMOST) combine mechanical sectioning with optical imaging. However, the “transparent” property distinguishes TESOS from fMOST. The TESOS approach is more like block reconstruction, where the final 3D stack is assembled from consecutive and overlapping image blocks rather than from sections. Such a strategy enables its flexible compatibility with various imaging systems, including light sheet/confocal/2-photon microscopies. A recently published fMOST system, array-fMOST, reduces sample preparation time by embedding brains directly in agarose, and also avoids possible morphological changes during tissue clearing [18]. However, it requires a self-made slicer and a customized imaging system. TESOS does not require specialized imaging setups, which simplifies the operational cost for general laboratories. Still, modifications are invariably required, including the MagMount device. In general, the different characteristics of these methods lead to different applicable scenarios.

The advancements in spatial transcriptomics and proteomics have increased the demand for 3D multiplex immunofluorescence and RNA in situ hybridization, along with their 3D detection [19,20]. Traditionally, these techniques are used on thin slices [21], which makes it challenging to scale them up for whole-mount samples for 2 main reasons: First, fluorophore-conjugated macromolecules are difficult to diffuse into thick samples, making the labeling process difficult and very time-consuming; second, during 3D imaging, fluorescent signals may weaken or become distorted in deeper regions due to insufficient transparency and refractive index mismatch, interfering with fluorescence colocalization analysis. When using optics with low NA for imaging cleared samples, the reduced axial resolution can lead to overlapping fluorescence signals at different *z* positions, further compromising the accuracy of colocalization assessments.

Therefore, using a high-NA objective to image thinner stacks at a time is the best way to gather data for 3D multiplex immunofluorescence and RNA in situ hybridization. The TESOS method and its 3D imaging pipeline can be a potential option. By employing high-NA objectives, both lateral and axial resolutions are enhanced, which improves the accuracy of colocalization assessments. Furthermore, the process of transparent embedding, imaging, and sectioning ensures that imaging maintains high quality and resolution, reducing the risk of misinterpreting results due to weak or distorted fluorescence signals in deeper regions.

The TESOS method shows satisfactory preservation of endogenous fluorescence after clearing and polymerization. For transgenic fluorescent samples, no additional fluorescent staining is needed to boost the signal. Linear channel unmixing method can be used to subtract autofluorescence from the target signal to improve both image quality and the signal-to-noise ratio. Meanwhile, the TESOS method is compatible with viral labeling and immunofluorescence staining, which further broadens its applicability, especially for primate samples. However, like other tissue clearing methods, the limited penetration of antibodies in immunofluorescence staining is still a challenge to be addressed. vDISCO utilizes nanobody for whole mouse body labeling [6]. Mai et al. [22] recently reported wildDISCO, which enables deep and homogeneous penetration of standard antibodies. Stochastic electrotransport proves to be able to rapidly disperse antibodies and such devices have already been commercialized. In the future, improved fluorescent probes with reduced size, innovative approaches to facilitate membrane permeabilization, and simplified devices to speed antibody diffusion combined with the TESOS method might further improve whole-tissue fluorescent labeling for large samples. On the other hand, viral labeling is a widely employed method for brain connectome and whole-body neural projection study. For long-range projections, it is suggested to inject high-titer adeno-associated virus (AAV) vectors containing Cre recombinase under a specific promoter into a cre-reporter mouse like Ai140 to get strong fluorescent protein expression within the axons. Brighter fluorescent proteins like mScarlet can be used in AAV design, especially for long-range or sparse labeling.

Detailed deciphering of organ anatomical architectures and connectivity is required to understand its function and dysfunction. The task is much more challenging for the study of nonhuman primates and even human tissues. Recent advances in imaging and single-cell projectome mapping technologies have greatly enriched our understanding in the organization principles of neural circuitry [23,24]. New whole-brain mapping pipelines have emerged to investigate structural interactions during brain diseases that minimize tissue distortion [25]. However, the imaging resolution and speed are to be further improved. As the need for brain connectome mapping and whole-body projection mapping increases, traditional tissue clearing methods designed for small samples with single-cell resolution imaging must be transformed. Combining transparent embedding with sectioning is likely the future direction for tissue clearing and 3D imaging. Future improvements for transparent embedding systems like TESOS may include (a) achieving greater transparency for better compatibility with light-sheet microscopy; (b) increasing gel strength to further minimize tissue deformation and improve stitching accuracy; and (c) automating the processes to lower labor costs.

To fully utilize the potential of innovative tissue clearing and transparent embedding techniques for acquiring structural data at the subcellular level, there is a need for microscopes that enable rapid, high-resolution imaging of large volumes. V-shaped light sheet setup will be especially effective for imaging large, thick transparently embedded samples. Additionally, there is a strong demand for computational methods for managing large-scale data, including multi-stack registration and stitching, 3D rendering, interactive data viewing and analysis, and large data storage. With advancements in imaging and data processing techniques, the transparent embedding method will become a powerful tool for achieving uniformly high-resolution images in neuroscience and other biomedical research.

## References

[1] Ueda HR, Erturk A, Chung K, Gradinaru V, Chedotal A, Tomancak P, Keller PJ. Tissue clearing and its applications in neuroscience. Nat Rev Neurosci. 2020;21(2):61–79. 31896771 10.1038/s41583-019-0250-1 PMC8121164

[2] Pan C, Cai R, Quacquarelli FP, Ghasemigharagoz A, Lourbopoulos A, Matryba P, Plesnila N, Dichgans M, Hellal F, Erturk A. Shrinkage-mediated imaging of entire organs and organisms using uDISCO. Nat Methods. 2016;13(10):859–867. 27548807 10.1038/nmeth.3964

[3] Tainaka K, Kubota SI, Suyama TQ, Susaki EA, Perrin D, Ukai-Tadenuma M, Ukai H, Ueda HR. Whole-body imaging with single-cell resolution by tissue decolorization. Cell. 2014;159(4):911–924. 25417165 10.1016/j.cell.2014.10.034

[4] Chung K, Wallace J, Kim S-Y, Kalyanasundaram S, Andalman AS, Davidson TJ, Mirzabekov JJ, Zalocusky KA, Mattis J, Denisin AK, et al. Structural and molecular interrogation of intact biological systems. Nature. 2013;497(7449):332–337. 23575631 10.1038/nature12107 PMC4092167

[5] Jing D, Zhang S, Luo W, Gao X, Men Y, Ma C, Liu X, Yi Y, Bugde A, Zhou BO, et al. Tissue clearing of both hard and soft tissue organs with the PEGASOS method. Cell Res. 2018;28(8):803–818. 29844583 10.1038/s41422-018-0049-z PMC6082844

[6] Cai R, Pan C, Ghasemigharagoz A, Todorov MI, Förstera B, Zhao S, Bhatia HS, Parra-Damas A, Mrowka L, Theodorou D, et al. Panoptic imaging of transparent mice reveals whole-body neuronal projections and skull–meninges connections. Nat Neurosci. 2019;22(2):317–327. 30598527 10.1038/s41593-018-0301-3 PMC6494982

[7] Tainaka K, Kuno A, Kubota SI, Murakami T, Ueda HR. Chemical principles in tissue clearing and staining protocols for whole-body cell profiling. Annu Rev Cell Dev Biol. 2016;32:713–741. 27298088 10.1146/annurev-cellbio-111315-125001

[8] Jing D, Yi Y, Luo W, Zhang S, Yuan Q, Wang J, Lachika E, Zhao Z, Zhao H. Tissue clearing and its application to bone and dental tissues. J Dent Res. 2019;98(6):621–631. 31009584 10.1177/0022034519844510 PMC6535919

[9] Wang F, Wan H, Ma Z, Zhong Y, Sun Q, Tian Y, Qu L, Du H, Zhang M, Li L, et al. Light-sheet microscopy in the near-infrared II window. Nat Methods. 2019;16(6):545–552. 31086342 10.1038/s41592-019-0398-7 PMC6579541

[10] Yoon S, Cheon SY, Park S, Lee D, Lee Y, Han S, Kim M, Koo H. Recent advances in optical imaging through deep tissue: Imaging probes and techniques. Biomater. Res. 2022;26(1):57. 36273205 10.1186/s40824-022-00303-4 PMC9587606

[11] Yi Y, Li Y, Zhang S, Men Y, Wang Y, Jing D, Ding J, Zhu Q, Chen Z, Chen X, et al. Mapping of individual sensory nerve axons from digits to spinal cord with the transparent embedding solvent system. Cell Res. 2024;34(2):124–139. 38168640 10.1038/s41422-023-00867-3 PMC10837210

[12] Ueda HR, Dodt H-U, Osten P, Economo MN, Chandrashekar J, Keller PJ. Whole-brain profiling of cells and circuits in mammals by tissue clearing and light-sheet microscopy. Neuron. 2020;106:369–387. 32380050 10.1016/j.neuron.2020.03.004 PMC7213014

[13] Ultra-long-working-distance multiphoton objective unlocks new possibilities for imaging. Nat Methods. 2024;21(1):24–25. 38129619 10.1038/s41592-023-02116-2

[14] Chen Y, Li X, Zhang D, Wang C, Feng R, Li X, Wen Y, Xu H, Zhang XS, Yang X, et al. A versatile tiling light sheet microscope for imaging of cleared tissues. Cell Rep. 2020;33(5): Article 108349. 33147464 10.1016/j.celrep.2020.108349

[15] Voigt FF, Reuss AM, Naert T, Hildebrand S, Schaettin M, Hotz AL, Whitehead L, Bahl A, Neuhauss SCF, Roebroeck A, et al. Reflective multi-immersion microscope objectives inspired by the Schmidt telescope. Nat Biotechnol. 2024;42(1):65–71. 36997681 10.1038/s41587-023-01717-8 PMC10791577

[16] Pacheco S, Wang C, Chawla MK, Nguyen M, Baggett BK, Utzinger U, Barnes CA, Liang R. High resolution, high speed, long working distance, large field of view confocal fluorescence microscope. Sci Rep. 2017;7(1):13349. 29042677 10.1038/s41598-017-13778-2 PMC5645379

[17] Yu C-H, Yu Y, Adsit LM, Chang JT, Barchini J, Moberly AH, Benisty H, Kim J, Young BK, Heng K, et al. The Cousa objective: A long-working distance air objective for multiphoton imaging in vivo. Nat Methods. 2024;21(1):132–141. 38129618 10.1038/s41592-023-02098-1 PMC10776402

[18] Chen S, Liu G, Li A, Liu Z, Long B, Yang X, Gong H, Li X. Three-dimensional mapping in multi-samples with large-scale imaging and multiplexed post staining. Commun Biol. 2023;6(1):148. 36737476 10.1038/s42003-023-04456-3 PMC9898531

[19] Wang X, Allen WE, Wright MA, Sylwestrak EL, Samusik N, Vesuna S, Evans K, Liu C, Ramakrishnan C, Liu J, et al. Three-dimensional intact-tissue sequencing of single-cell transcriptional states. Science. 2018;361(6400):eaat5691. 29930089 10.1126/science.aat5691 PMC6339868

[20] Tao Y, Zhou X, Sun L, Lin D, Cai H, Chen X, Zhou W, Yang B, Hu Z, Yu J, et al. Highly efficient and robust π-FISH rainbow for multiplexed in situ detection of diverse biomolecules. Nat Commun. 2023;14(1):443. 36707540 10.1038/s41467-023-36137-4 PMC9883232

[21] Moffitt JR, Bambah-Mukku D, Eichhorn SW, Vaughn E, Shekhar K, Perez JD, Rubinstein ND, Hao J, Regev A, Dulac C, et al. Molecular, spatial, and functional single-cell profiling of the hypothalamic preoptic region. Science. 2018;362(6416):eaau5324. 30385464 10.1126/science.aau5324 PMC6482113

[22] Mai H, Luo J, Hoeher L, Al-Maskari R, Horvath I, Chen Y, Kofler F, Piraud M, Paetzold JC, Modamio J, et al. Whole-body cellular mapping in mouse using standard IgG antibodies. Nat Biotechnol. 2024;42(4):617–627. 37430076 10.1038/s41587-023-01846-0 PMC11021200

[23] Park J, Wang J, Guan W, Gjesteby LA, Pollack D, Kamentsky L, Evans NB, Stirman J, Gu X, Zhao C, et al. Integrated platform for multiscale molecular imaging and phenotyping of the human brain. Science. 2024;384(6701):eadh9979. 38870291 10.1126/science.adh9979 PMC11830150

[24] Nudell V, Wang Y, Pang Z, Lal NK, Huang M, Shaabani N, Kanim W, Teijaro J, Maximov A, Ye L. HYBRiD: Hydrogel-reinforced DISCO for clearing mammalian bodies. Nat Methods. 2022;19(4):479–485. 35347322 10.1038/s41592-022-01427-0 PMC9337799

[25] Zhu J, Liu X, Liu Z, Deng Y, Xu J, Liu K, Zhang R, Meng X, Fei P, Yu T, et al. SOLID: Minimizing tissue distortion for brain-wide profiling of diverse architectures. Nat Commun. 2024;15:8303. 39333107 10.1038/s41467-024-52560-7 PMC11436996

